# Gene Order Phylogeny of the Genus *Prochlorococcus*

**DOI:** 10.1371/journal.pone.0003837

**Published:** 2008-12-03

**Authors:** Haiwei Luo, Jian Shi, William Arndt, Jijun Tang, Robert Friedman

**Affiliations:** 1 Department of Biological Sciences, University of South Carolina, Columbia, South Carolina, United States of America; 2 Department of Computer Science and Engineering, University of South Carolina, Columbia, South Carolina, United States of America; University of Stellenbosch, South Africa

## Abstract

**Background:**

Using gene order as a phylogenetic character has the potential to resolve previously unresolved species relationships. This character was used to resolve the evolutionary history within the genus *Prochlorococcus*, a group of marine cyanobacteria.

**Methodology/Principal Findings:**

Orthologous gene sets and their genomic positions were identified from 12 species of *Prochlorococcus* and 1 outgroup species of *Synechococcus*. From this data, inversion and breakpoint distance-based phylogenetic trees were computed by GRAPPA and FastME. Statistical support of the resulting topology was obtained by application of a 50% jackknife resampling technique. The result was consistent and congruent with nucleotide sequence-based and gene-content based trees. Also, a previously unresolved clade was resolved, that of MIT9211 and SS120.

**Conclusions/Significance:**

This is the first study to use gene order data to resolve a bacterial phylogeny at the genus level. It suggests that the technique is useful in resolving the Tree of Life.

## Introduction

Comparisons of genomes reveal difference in the order of genes. This provides a phylogenetic character to resolve species relationships and complements the standard approach of using the nucleotide as the character of interest. Genes are rearranged in the genome by evolutionary events such as inversion, transposition, and inverted transposition, collectively called genome rearrangements [1]–[3]. Since these are rare events, a phylogeny inferred from gene rearrangements has the potential to resolve ancient phylogenetic relationships [2]. Consequently, gene order data has been used in phylogenetic reconstructions of mitochondrion and chloroplast genomes [4]–[6] as well as bacterial genomes [7]. With the increasing number of whole genome sequences and the development of new algorithms, gene order data presents an accessible means to reconstruct species phylogenies [7].

*Prochlorococcus* is a genus of photosynthetic marine cyanobacteria which accounts for nearly one-half of the photosynthetic biomass and primary production in tropical and subtropical oceans [8], [9]. Past phylogenetic studies of *Prochlorococcus* has mainly relied upon nucleotide sequence data [10]–[12]. However, some key parts of the topology were unresolved. For example, whether SS120 and MIT9211 form a monophyletic group remained unknown [11]. In this study, we use alternative approaches to investigate the *Prochlorococcus* phylogeny and better resolve the topology.

Currently, twelve whole genome sequences in the genus *Prochlorococcus* are available and provide an opportunity to study the evolution of this organism from a genomic perspective. In this study, we use inversion and breakpoint distances to reconstruct the phylogeny of the genus *Prochlorococcus* and to resolve a controversial node with statistical confidence.

## Methods

### Genome annotation

The whole genomic DNA sequences of the 12 *Prochlorococcus* genomes and the *Synechococcus* WH8102 genome were downloaded from NCBI and re-annotated by the RAST Server [13]. The RAST Server identified protein-encoding, rRNA and tRNA genes using subsystem technology and formatted the results as a Genbank file [13]. Using Perl scripts, this file was parsed for the predicted protein-coding sequences and their corresponding genomic positions.

### Ortholog identification

The predicted protein sequences from these 13 genomes were pooled, and then the BLASTCLUST software [14] was used to cluster sequences based upon their similarity. BLASTCLUST is a two-step procedure where homologous genes are identified by pairwise similarity and then clustered into gene families by the single-linkage method. The criteria used to find pairwise matches were more than 30% sequence similarity across a minimum of 50% of their lengths. For the gene families with paralogs, additional BLASTCLUST procedures with higher stringency were implemented to discover the true orthologs. This process was repeated to recover all putative protein-coding orthologous genes shared by these 13 genomes.

### Gene order generation

Perl scripts were written to extract the positions of all protein-coding regions. The order of orthologs in each genome was determined based upon their starting position and strandedness. Each genome was coded as an ordered set of signed genes where sign indicates strandedness [2].

### Gene order phylogenetic reconstruction

GRAPPA [1], [15] was used to compute the pairwise inversion and breakpoint distances from the gene order data. This software outputs a distance matrix. Then the FastME [16] program constructed the inversion and breakpoint distance-based phylogenetic trees. The tree topologies were displayed by MEGA4 [17]. To calculate the statistical reliability of the branches of the phylogeny, we applied a jackknife resampling technique that randomly removed 50% of the initial orthologous gene sets. We generated 100 jackknife random samples and acquired 100 matrices for both inversion and breakpoint distances. These 100 matrices were imported into the FastME program to obtain 100 inversion and breakpoint distance-based trees. Finally, the CONSENSE program in the PHYLIP software package [18] was used to obtain a majority rule consensus tree with the numbers at each node representing the percentage that the clade defined by that node appears in the 100 jackknife trees. These values were assigned to the nodes of the initial gene order tree.

## Results and Discussion

### Orthologs shared among Prochlorococcus and Synechococcus WH8102 genomes

Reconstruction of inversion and breakpoint distance-based phylogenies depend on finding shared orthologous genes among the organisms of interest. The BLASTCLUST procedure revealed 1131 orthologous genes which are shared by all 13 genomes, the 12 *Prochlorococcus* and the outgroup *Synechococcus* WH8102. These 1131 orthologous genes represent the core genome. This count of orthologous genes is lower than the 1273 reported previously [11], but this latter study had no outgroup.

### Sequence-based and gene-content based phylogeny of Prochlorococcus

Based on the pigment composition as well as their phylogenetic relationships, *Prochlorococcus* cells are classified into high light (HL) adapted and low light (LL) adapted ecotypes [12]. To date, 12 *Prochlorococcus* genomes have been sequenced, which consist of 6 HL and 6 LL strains.

**Figure 1:**
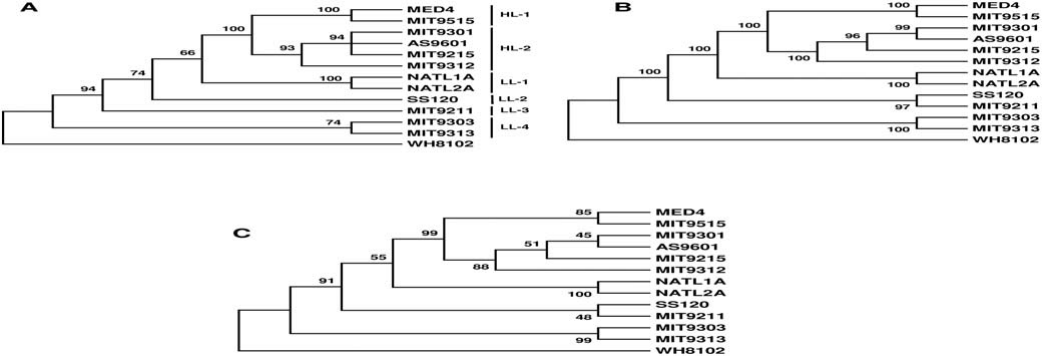
Phylogeny of 12 *Prochlorococcus* genomes inferred from (A) 16S rRNA, (B) random concatenation of 100 protein sequences sampled from core genome, and (C) concensus tree of all core genes. Trees were reconstructed by neighbor-joining (A), maximum parsimony (B), and maximum parsimony (C) methods. The trees are reprints from Kettler [11]. HL, high light adapted strains; LL, low light adapted strains.

The usual approach of using 16S rRNA and protein-coding genes has been applied to reconstructing the relationships within *Prochlorococcus*. In the 16S rRNA tree and the consensus tree inferred from single core genes, the 6 HL genomes form a monophyletic cluster, while 6 LL genomes are paraphyletic (Fig. 1A & 1C). In addition, these two trees show that the 6 HL genomes form 2 separate clades, with MED4 and MIT9515 comprising one cluster and the remaining 4 HL genomes forming the other cluster.

Although a single gene tree has been used extensively to estimate the species tree, evidence has been shown that gene trees may differ in topology from each other and the true tree [19]. Also, it is especially difficult to use gene trees to represent the prokaryotic species phylogeny, as it has been documented that lateral gene transfer (LGT) occurs among prokaryotic genomes, and LGT may obscure the phylogenetic signal [20]. Although it was believed that such reconstruction is still possible if a group of core orthologous genes is available which is not affected by LGT, it is difficult to obtain such an orthologous gene set that is refractory to LGT [20], [21]. Consequently, we do not know the fraction of truly orthologous genes that were used to reconstruct the consensus tree of *Prochlorococcus* (Fig. 1C).

A recent study proposed that the “Tree of Life” may be resolved by concatenation of 31 orthologs occurring in 191 species [22], and an analogous approach has been applied to infer *Prochlorococcus* phylogeny by random concatenation of 100 protein sequences sampled from the *Prochlorococcus* core genome [11]. The concatenation-based phylogeny is consistent with the above relationships shown in the 16S rRNA tree and the consensus tree (Fig. 1A, 1B & 1C).

Recently, it has been proposed that gene content is a phylogenetically informative character in species phylogeny reconstruction [23]. The gene-content based phylogeny has also been reconstructed for the genus *Prochlorococcus*, which shows the same topology as the gene concatenation tree [11].

Among the 6 LL genomes, all of the above three sequence-based phylogenies and the gene-content tree show that NATL1A and NATL2A form a cluster, and MIT9303 and MIT9313 form another cluster. However, these trees differ in regard to whether SS120 and MIT9211 form a separate cluster. Though the concatenation-based and gene-content based phylogeny supported the monophyly of these two organisms (Fig. 1B), the consensus tree did not show sufficient support and the 16S rRNA supported alternative topologies for this node (Fig. 1A & 1C [11]). In the sequence-based consensus tree, values for each node represent fraction of genes supporting each node, and it is apparent that the SS120/MIT9211 node lacks sufficient statistical support (Fig. 1C), and in the 16S rRNA tree they do not cluster. In other words, the 16S rRNA phylogeny supports four separate clades for the 6 LL genomes. The 16S rRNA phylogeny has been frequently cited and discussed elsewhere [11], [24], [25].

Although it has been shown that phylogenetic reconstruction based upon concatenation of multiple orthologous genes can generate a more accurate tree than a consensus of multiple gene trees, concatenated alignments may severely reinforce the systematic errors accompanied with some individual genes, which can be erroneously represented by high bootstrap values [26]. Therefore, it would be desirable to investigate the questionable node in the *Prochlorococcus* phylogeny using other models independent of sequence-based methods.

### Gene order phylogeny of Prochlorococcus

Recently, gene order phylogenies have been gaining ground [4]–[7]. Distance-based reconstruction of gene order phylogeny can be implemented by computing breakpoint and inversion distances. Breakpoint distance is the number of gene adjacencies that are present in one genome but absent from the other genome, hence breakpoint distance describes the dissimilarity of the gene order between two genomes [27]. Inversion distance is calculated from the minimum number of inversion events that are required to convert one genome to the other [27]. As has been shown, both breakpoint and inversion distances may underestimate the true evolutionary distance, while a distance-correction algorithm, called empirically derived estimator (EDE), outperforms the other two methods by correcting the minimum inversion distance between the two genomes [27].

Alteration of gene order implemented by inversion, transposition, and inverted transposition was deemed as rare events, so it has been suggested that gene order phylogeny reconstruction is able to resolve deep phylogenetic relationships [27]. Indeed, gene order data was successfully applied to reconstruct phylogeny of 30 genomes of Gamma-Proteobacteria [7]. However, no studies have been reported regarding the application of gene order data in phylogenetic reconstruction of shallow relationships, such as at the genus level. We are fulfilling this gap by illustrating that gene order based phylogeny is able to resolve a controversial node regarding the position of MIT9211 and SS120 in the phylogeny of the genus *Prochlorococcus*.

**Figure 2:**
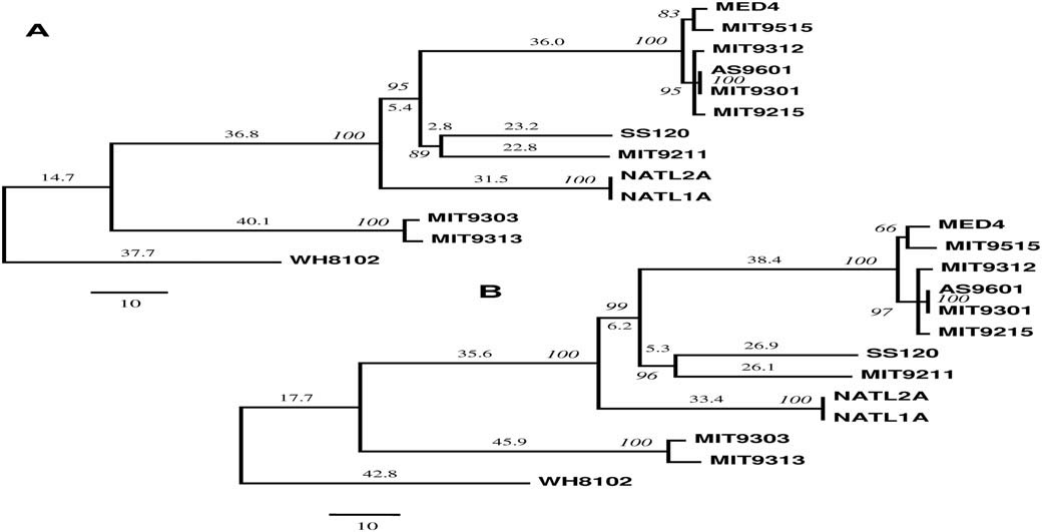
Phylogeny of 12 *Prochlorococcus* genomes inferred from (A) an empirically derived estimator (EDE) inversion distance and (B) a breakpoint distance matrix tree. Values at nodes show the number of times the clade defined by that node appeared in the 100 jacknife trees. Values above branches and the scale bar show number of genome rearrangement events. The *Synechococcus* WH8102 is used as an outgroup.

The inversion distance-based tree has the same topology as the breakpoint distance-based tree (Fig. 2). There was also a strong correlation between inversion and breakpoint distances (R = 0.99), indicating that inversions are the primary events causing the breakpoints in *Prochlorococcus* genomes. The inversion and breakpoint distance-based trees are generally consistent with the sequence-based and gene-content based trees, showing that 6 HL genomes form a monophyletic group, while 6 LL genomes do not, and that the 6 HL genomes comprise two separate clades. Regarding the 6 LL genomes, inversion and breakpoint distance-based phylogenies show that NATL1A and NATL2A, as well as MIT9313 and MIT9303 form two separate clusters, which is congruent with all sequence-based trees. However, the two gene order phylogenies provided strong evidence that MIT9211 and SS120 form a separate cluster by showing high jackknifing value support. It appears that gene order phylogeny is a potent tool to resolve controversial nodes generated by sequence-based methods.

Together with the observation that the gene order tree is generally congruent with sequence-based and gene-content based cladograms, we conclude that gene order data is useful in phylogenetic reconstruction of closely related bacteria.

### Proposed phylogeny of Prochlorococcus

Though gene order approach is powerful in resolving controversial phylogenetic relationships, there are some limitations for the application of gene order to phylogenetic reconstruction of closely related organisms. We notice that the phylogenetic relationship of four organisms, including MIT9312, MIT9215, MIT9301, and AS9601, was not resolved in the gene order tree. We further note that the inversion and breakpoint distance between each pair of the four organisms were very low (<10 events), suggesting that few genes were altered in their genomic positions. When 50% of the common orthologous genes were removed in the 100 jackknifing tests, those few phylogenetically informative genes were likely removed many times, resulting in collapse of the topology of those four organisms. In addition, the inversion and breakpoint distance-based trees show that the MIT9211/SS120 clade is closer to the 6 HL genomes than the NATL1A/NATL2A clade with high jackknifing value support, while the sequence-based trees show that the latter clade is closer to the HL species/strains than the former one. These two hypotheses are not yet resolved.

**Figure 3:**
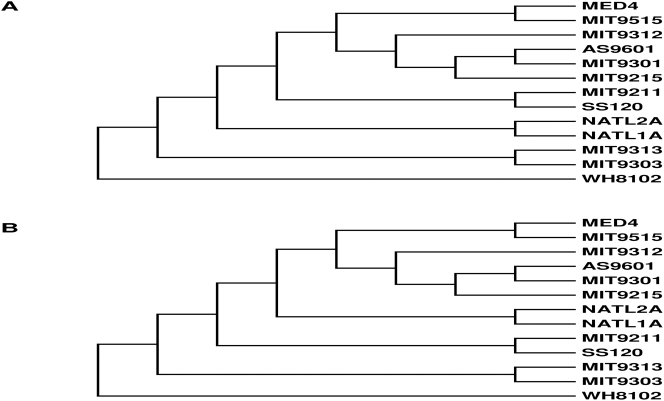
Proposed phylogeny of 12 *Prochlorococcus* genomes by integrating gene order phylogeny as well as phylogeny inferred from random concatenation of 100 core genes. (A) MIT9211/SS120 clade is closer to the HL species (B) NATL1A/NATL2A clade is closer to the HL species.

We integrated the phylogenetic reconstruction of *Prochlorococcus* from gene order data as well as concatenated alignments, and proposed two alternative phylogenies of the genus *Prochlorococcus* in Figures 3A and 3B.
